# Real‐time detection of cerebrospinal fluid with bioimpedance needle in paediatric lumbar puncture

**DOI:** 10.1111/cpf.12697

**Published:** 2021-03-20

**Authors:** Harri Sievänen, Juho Kari, Sanna Halonen, Timo Elomaa, Outi Tammela, Hanna Soukka, Vesa Eskola

**Affiliations:** ^1^ Injeq Oy Tampere Finland; ^2^ Department of Pediatrics Tampere University Hospital Tampere Finland; ^3^ Tampere Center for Child Health Research Tampere University Tampere Finland; ^4^ Department of Pediatrics and Adolescent Medicine Turku University Hospital Turku Finland; ^5^ University of Turku Turku Finland

**Keywords:** Bioimpedance measurement, infant, neonatal, spinal tap, tissue detection

## Abstract

**Background:**

Lumbar puncture is a common clinical procedure that can occasionally be difficult. Various needle guidance methods can facilitate performing this procedure, but at the expense of special expertise, equipment and facility. In the present study, we evaluated the clinical feasibility of a novel bioimpedance needle system regarding its ability to detect cerebrospinal fluid (CSF) in paediatric lumbar punctures.

**Methods:**

We performed 40 lumbar puncture procedures using the bioimpedance needle system in 37 paediatric patients, aged from 0 days to 17 months, as a part of their prescribed examinations in two university hospitals. The bioimpedance needle is similar to a conventional 22G cutting‐edge spinal needle with a stylet, except the needle and stylet are configured as a bipolar electrode with high spatial resolution. The system measures in real‐time when the needle tip reaches the subarachnoid space containing CSF. The procedure was considered successful when the erythrocyte count was determined from the obtained CSF sample.

**Results:**

Subarachnoid space was verifiably reached in 28 out of 40 procedures (70%). Bioimpedance needle system detected CSF in 23 out of these 28 successful procedures (82%) while failed in 3 out of 28 procedures (11%). No adverse events were reported.

**Conclusion:**

Bioimpedance spinal needle system was found clinically feasible in paediatric lumbar punctures, and it may offer an objective and simple means to detect the time point when the needle tip is in contact with the cerebrospinal fluid.

## 1 INTRODUCTION

Lumbar puncture is an established clinical procedure to get a diagnostic sample of cerebrospinal fluid (CSF) or to inject anaesthetics or intrathecal therapy into the subarachnoid space (Bonadio et al, 2014; Schulga et al., 2015; Srinisavan et al., 2012). Laboratory analyses of CSF can reveal many severe diseases of the central nervous system, including infections and inflammatory, haemorrhagic or neoplastic diseases.

Lumbar puncture is occasionally challenging to perform. Advancing a thin, flexible needle into the spinal canal through narrow spaces between lumbar bony structures without seeing the location of the needle tip or its direction while interpreting the tactile feedback from penetrated tissues requires clinical experience and touch to the patient's spinal anatomy (Boon et al., 2004). For small neonates and infants, the performance is often more difficult. Traumatic lumbar punctures with blood in the CSF sample or failed punctures at the first attempt are common in paediatrics (Baxter et al., 2006; Glatstein et al., 2011; Greenberg et al. 2008; Nigrovic et al., 2007). More than a half of paediatric procedures may be compromised (Auerbach et al., 2018; Kessler et al., 2013; Neal et al., 2017). Excess blood in the CSF sample can essentially reduce the sample quality and increase diagnostic uncertainty (Muthusami et al., 2017). In children with acute lymphoblastic leukaemia, the success of lumbar puncture is particularly important to prevent blasts from entering the spinal canal during the first diagnostic puncture and to allow proper delivery of intrathecal chemotherapy during the subsequent treatment punctures (Shaikh et al., 2014).

Various needle guidance methods can facilitate performing the lumbar puncture. Fluoroscopic real‐time X‐ray imaging and needle guidance can be effectively employed in the most challenging cases (Cauley 2015), and this method can reduce puncture‐related complications (Rodriguez et al., 2016). However, special expertise, equipment and facility are required, let alone the radiation exposure to the patient and personnel. Computed tomography is also an option (Hudkins et al., 2017), but its clinical utility is compromised by the same issues as with fluoroscopy. Ultrasound does not involve X‐ray radiation, and the ultrasound guidance can improve the success rate of performing a lumbar puncture and reduce the number of attempts, total puncture time and complications in neonates and infants (Muthusami et al., 2017; Olowoyeye et al., 2019). Again, besides the equipment, this method requires the physician's one hand to use the ultrasonic transducer and dedicated skills on mastering the transducer and interpreting the ultrasound image in real time. There is thus room for a feasible and simple method for the needle guidance that would not essentially alter the standard procedure of lumbar puncture while being of clinical value.

Recently, the use of a conventional spinal needle configurated as a bioimpedance electrode yielded a sensitivity of 100% and a specificity of 81% in detecting CSF in adult lumbar punctures for spinal anaesthesia (Halonen et al., 2017). These clinical findings implied that the novel bioimpedance needle system can detect the time point when the needle tip reaches the subarachnoid space and gets in contact with CSF, and facilitate performing the procedure. The present study extended the target group to paediatric patients and evaluated the feasibility and performance of the bioimpedance needle system in lumbar punctures of neonates and infants as well.

## 2 MATERIALS AND METHODS

### 2.1 Study design

We conducted this medical device investigation in Tampere and Turku University Hospitals (Finland) during the years 2016 – 2018. The study comprised two stages: first, to get first‐hand clinical experience in performing lumbar punctures with the novel bioimpedance needle system in small paediatric patients and to fine‐tune the tissue detection algorithm if considered relevant, and secondly, to evaluate the performance of the system in detecting CSF in paediatric lumbar punctures.

### 2.2 Patients

A patient was eligible for the study if his/her age was less than 18 months, and a lumbar puncture was required for getting a diagnostic CSF sample, or the procedure was a part of the patient's prescribed treatment plan. Written informed consent was obtained from patients’ parents before the study. The study protocol was approved by the Ethics Committee of Tampere University Hospital District. Also, the national competent authority (National Supervisory Authority of Health and Welfare (Valvira), Helsinki, Finland) was notified about this study before commencing it. The study was carried out following the Declaration of Helsinki and Good Clinical Practice. The study is registered in ClinicalTrials.gov (NCT02792660).

Exclusion criteria were a skin infection around the puncture area, bleeding tendency with thrombocytopenia, acute bleedings and problems with haemostasis, unstable haemodynamics with low blood pressure and need of inotropic agents, increased intracranial pressure with bulging fontanelles, use of ventriculoperitoneal shunt, previous back surgery or known spinal abnormalities.

### 2.3 Lumbar puncture

In the Tampere hospital, all lumbar punctures were performed by an experienced paediatrician (VE), whereas in the Turku hospital, according to the hospital's practice of medical training, yet unexperienced specializing physicians performed the punctures under the supervision of an experienced paediatrician (HSo), who completed the procedure if needed.

Paediatric lumbar punctures were done according to hospitals’ standard practices without general anaesthesia or sedation. For each patient, topical anaesthetic cream was applied to the puncture site and adequate time was given for the medication to take effect. Oral sucrose was used in neonatal patients before the procedure and care was taken that the patients had received paracetamol before the puncture.

The criterion for a successful lumbar puncture procedure was that a proper CSF sample was obtained and the erythrocyte count (i.e. the number of red blood cells per volume) was analysed. The erythrocyte count in the sample was determined with standard cytometric methods in the hospital laboratory. In all patients and both hospitals, the third vial of the CSF sample was used for the haematological analysis.

### 2.4 Bioimpedance needle system

Instead of a conventional spinal needle, a novel bioimpedance needle system was used in lumbar punctures. The bioimpedance needle is a conventional Quincke‐type spinal needle (size 22G and length 40 mm) with a stylet configured as a bioimpedance electrode (IQ‐Tip^TM^ Spinal Needle, Injeq Oy, Tampere, Finland, www.injeq.com). The stylet is electrically isolated from the needle body so that the needle tip provides a bipolar electrode and allows a highly localized, real‐time bioimpedance measurement of the tissue at the needle tip (Halonen et al., 2019). The stylet can be similarly removed and reinserted as with the conventional spinal needle.

**FIGURE 1:**
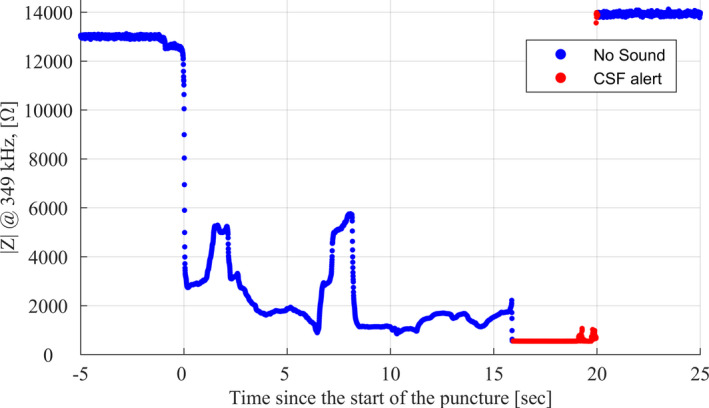
Measured impedance curve as a function of time during a successful lumbar puncture of a 1‐day‐old male neonate weighing 3·4 kg. The blue curve indicates the period when there was no alarm, and the red curve indicates the period, when the system gave an alarm and CSF was correctly detected

The stylet is connected to a bioimpedance analyser via a sterile, flexible coaxial cable, which all together make up the bioimpedance needle system (Injeq IQ‐Tip^TM^ System). The analyser employs a one millisecond long, proprietary binary pulse sequence in which most of the signal power is located at few specified frequencies within a range from 1 kHz to about 350 kHz. This low‐power signal (compliant with the medical safety standard IEC 60601‐1) is continuously transmitted to the needle as an excitation signal. Bioimpedance is simultaneously measured with the needle tip electrode within less than a 1 mm^3^ volume of surrounding tissues and calculated from discrete Fourier transformations of voltage and current signal values at the specified frequencies (Halonen et al., 2019). A bioimpedance curve measured during one paediatric lumbar puncture is shown for illustration in Figure 1. Calculated bioimpedance values are employed for tissue classification at a 200 Hz rate. This means that the tissue classification is performed virtually in real‐time (at every 5^th^ ms) with high spatial sensitivity (<1 mm^3^).

During the first stage of the study, the initial tissue classification algorithm was made slightly more sensitive, but in the second stage, the algorithm was kept fixed, and no further modifications were made to the algorithm.

### 2.5 Assessment of CSF detection accuracy

By design, the bioimpedance analyser is intended to give an audio‐visual alarm when the needle tip reaches CSF in the subarachnoid space. After the alarm and upon clinical judgement by the performing physician, he/she could either remove the stylet and check for CSF flow through the needle or continue the puncture. Irrespective of the preceding alarm or not, the physician could remove the stylet and check for CSF flow solely as per his/her clinical judgement of the instant location of the needle tip. The correct anatomic location of the needle tip in the subarachnoid space and the presence of CSF was verified by CSF flow and sampling after the stylet removal.

Immediately after completing the procedure, the physician evaluated and recorded the system performance on the patient's Case Report Form (CRF) in terms of the perceived CSF detection alarms and consequent or independent actions. Also, potential safety issues and adverse events during the procedure were inquired.

Raw impedance data collected during the procedure were stored in the analyser memory for further offline analysis. Results from the offline analysis were used for independent verification of the information the physicians recorded on the patients’ CRFs after completing their procedures. The physician's notes on the CRF served as the primary data regarding the system performance.

The physician may have described the system performance on the patient's CRF with the following indicators:

- correct detection, when the system alarmed and there was CSF flow after the stylet removal and the sample was analysed,
- false detection, when the system alarmed but there was no CSF flow, or the obtained sample was excessively blood‐tinged and the erythrocyte count was not determined,
- missed detection, when the system did not alarm but there was CSF flow after the stylet removal and the erythrocyte count was determined,
- no false detection, when the system did not alarm and there was no CSF flow after any stylet removal.

If any of the stylet removals resulted in CSF flow and the CSF sample was analysed in the laboratory verifying that the subarachnoid space was properly reached, the following four combinations of performance indicators were possible:

- correct detection and no false detection,
- false detection and correct detection,
- false detection and missed detection,
- missed detection and no false detection.

If the subarachnoid space was not verifiably reached, either false detection or no false detection was the only possible performance indicators.

The CSF detection accuracy and false detection rate were used as the clinical performance outcomes of the bioimpedance needle system. The CSF detection accuracy and the false detection rate were calculated with the following equations:

$$CSF\;detection\;accuracy\;\left(\%\right)\;=\;100∙\frac{N\;of\;Correct\;detections}{N\;of\;Correct\;detections\;+\;N\;of\;Missed\;detections}\%$$

$$False\;detection\;rate\;\left(\%\right)\;=\;100∙\frac{N\;of\;False\;detections}{N\;of\;False\;detections\;+\;N\;of\;No\;false\;detections}\%$$

where N denotes the total number of recorded performance indicators on the patients’ CRFs.

Mean values of these clinical performance outcomes are given as descriptive data.

## 3 RESULTS

The first stage of the study was performed in Tampere hospital only, and it comprised 10 procedures of nine patients aged from 2 days to 17 months. Four patients were neonates (younger than or equal to 28 days). The range of patients’ body weight was from 3·5 to 11·4 kg. The second stage was performed both in Tampere and Turku hospitals and comprised 30 procedures (15 in each hospital) of 28 patients aged from 0 days (including one preterm baby) to 6 months. Twenty‐five were neonates. The range of patients’ body weight was from 1·4 to 7·8 kg.

In the first stage of the study, subarachnoid space was verifiably reached in seven out of 10 procedures (70%), all in the first attempt. In these successful punctures, CSF was detected in all seven cases without false detections. In the three unsuccessful procedures, excessively bloody fluid was obtained after the stylet removal and the erythrocyte count was not determined from these samples.

In the second stage of the study, subarachnoid space was verifiably reached in 21 out of 30 procedures (70%). In these successful 21 procedures, CSF was detected in 16 out of 21 (76%) and there were three false detections (14%). In eight of the nine unsuccessful punctures, excessively bloody fluid was obtained after the stylet removal and the erythrocyte count was not determined; in one case, there was no flow observed.

**FIGURE 2:**
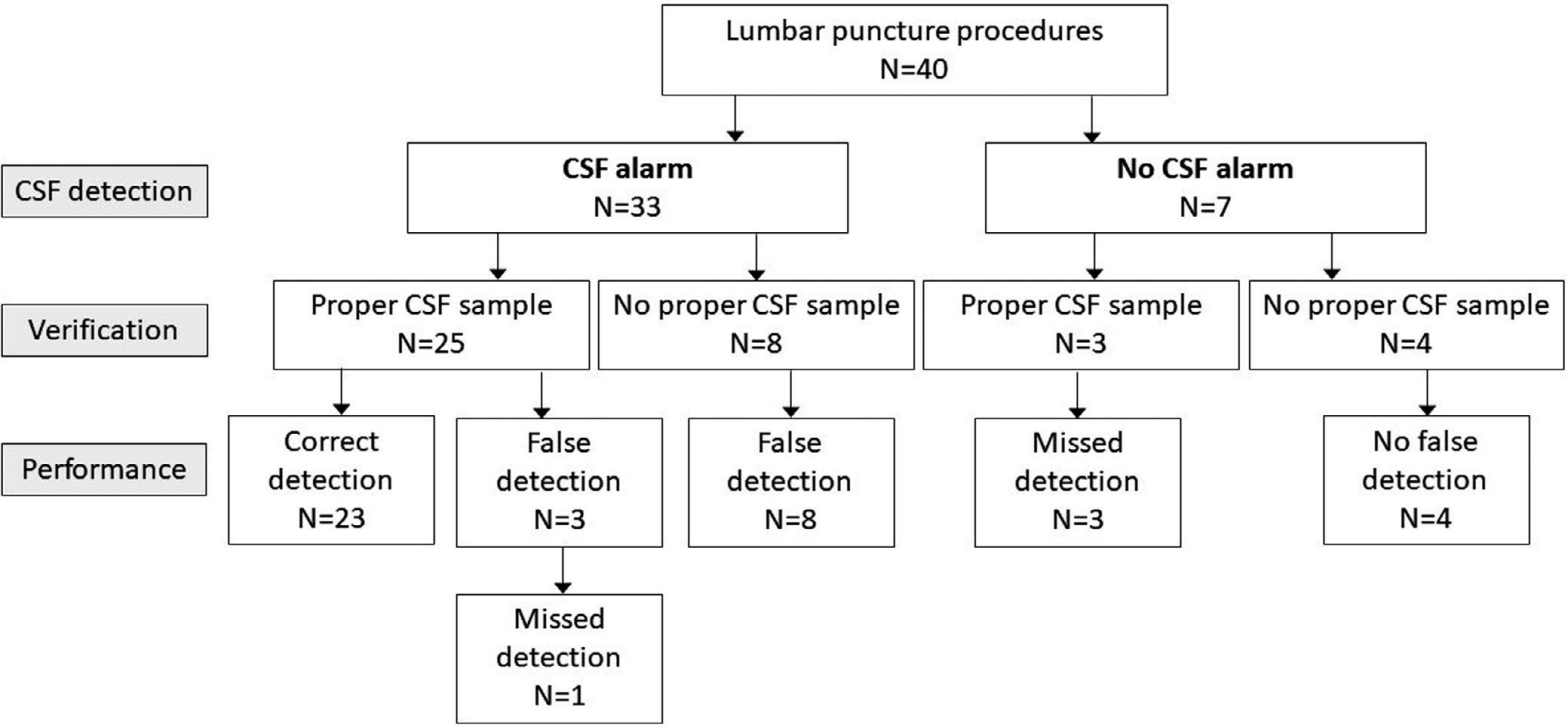
Flow chart of performance indicators observed in 40 lumbar puncture procedures. Verification of the CSF detection was based on getting a proper CSF sample from which the erythrocyte count was determined. Performance indicators for each procedure are also illustrated in Figure 3

**FIGURE 3:**
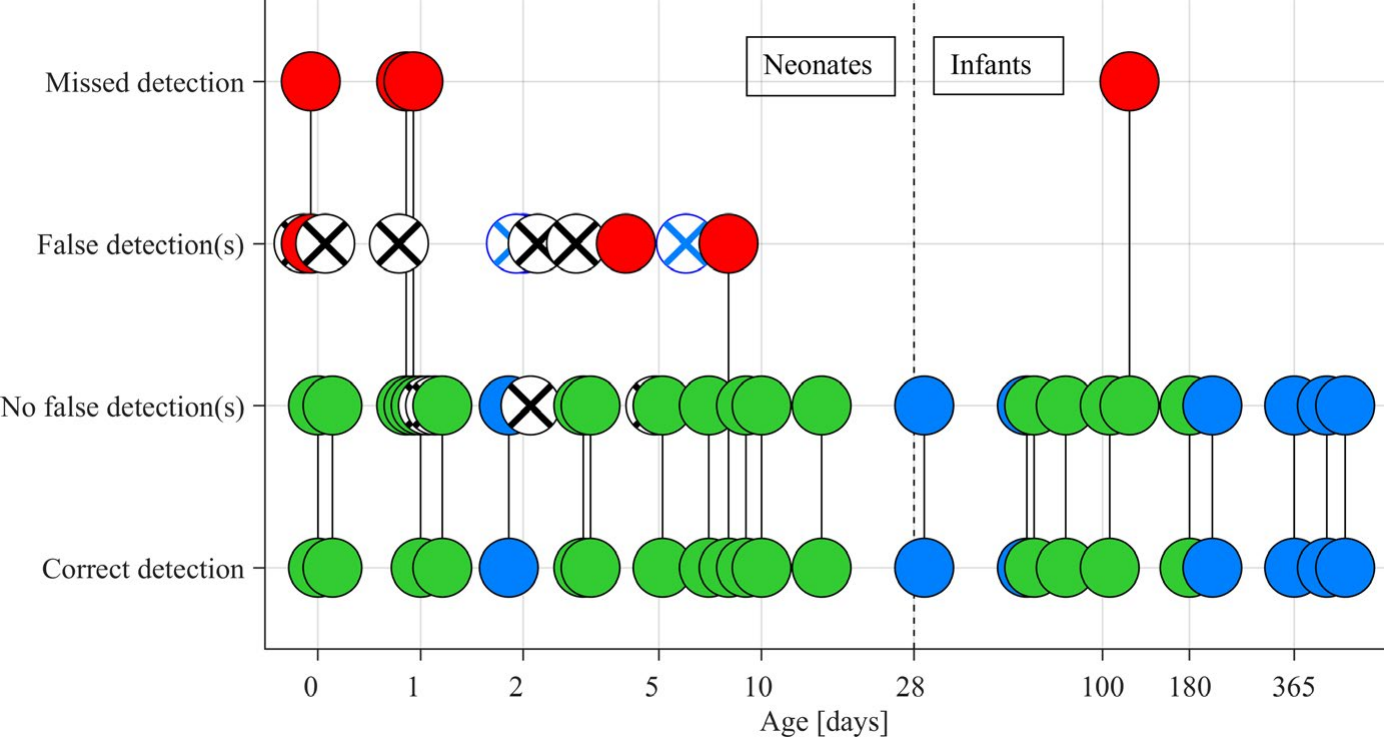
Performance indicators observed in 40 lumbar puncture procedures plotted against the patient's age (N.B., logarithmic scale of the x‐axis for clarity of presentation). Patients are divided into neonates and infants by their age. The 28 procedures with successfully obtained CSF samples are indicated by solid circles. The 12 procedures when the CSF sample was not successfully obtained are indicated by crossed open circles (blue or black). Blue colour denotes the data that was obtained from the first stage of the study, while the green/red/black colours denote the data obtained from the second stage of the study. Data points from the same patient are connected by a solid line as appropriate

In the pooled sample of both study stages, subarachnoid space was verifiably reached in 28 out of 40 procedures (70%). Bioimpedance needle system detected CSF in 23 out of 28 successful procedures (82%) while failed in 3 out of 28 procedures (11%). Performance indicators observed in all 40 procedures are depicted in Figure 2. in the form of a flow chart and illustrated in Figure 3 as a function of the patient's age.

**FIGURE 4:**
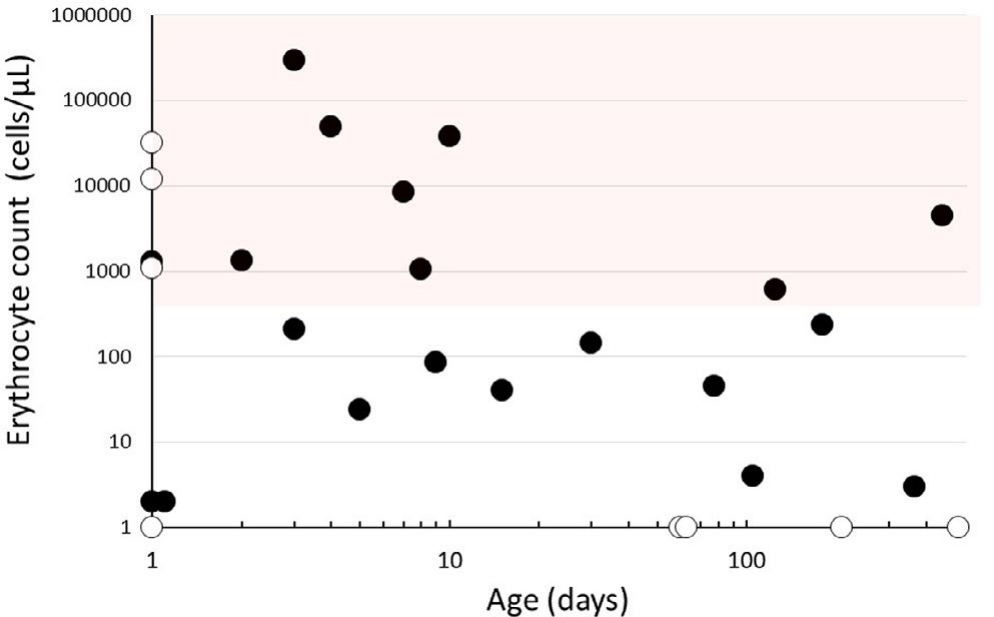
Erythrocyte counts from the 28 successful lumbar puncture procedures plotted against the patient's age (N.B., logarithmic scales of both axes for clarity of presentation). Because the logarithm of zero is undefined, open circles on the x‐axis indicate zero erythrocytes, and open circles on the y‐axis indicate the age of zero days. The shaded area indicates the region where the presence of blood in the CSF sample may be visual according to Shah et al., (2003)

Erythrocyte counts obtained from the 28 successful procedures are plotted against the patient's age in Figure 4. The range of laboratory‐evaluated erythrocyte counts was from 0 to 300 000 cells/µl.

Reinsertion of the spinal needle was needed only in four out of 40 procedures (10%). In one case, the second attempt was successful, but in the two other cases, excessively bloody fluid was again obtained. In one case, no CSF sample was obtained. No adverse events were reported during the 40 procedures.

The face‐to‐face comparison between the CRF data and independent offline analysis of the raw bioimpedance data indicated agreement in 38 out of 40 punctures (95%). In one case, the physician seemed to have disregarded the alarm although there was CSF flow after the stylet removal. In the other case, there was no CSF flow after the alarm, but after a small adjustment of the needle, CSF flow occurred.

## 4 DISCUSSION

The novel bioimpedance needle system was found feasible to perform lumbar punctures of neonates and infants. Its accuracy in detecting CSF was satisfactory although not quite comparable to the high accuracy observed in adult lumbar punctures using the same system. In the present paediatric procedures, the CSF detection accuracy was 82%, while in adults, it was 100% (Halonen et al., 2017). Corresponding false detection rates were 11% and 19%. The somewhat poorer performance in paediatric procedures could be expected because of smaller anatomy and different tactile feedback from neonatal tissues, and poorer ability of neonates and infants to stay still during the procedure (Bonadio et al, 2014; Schulga et al., 2015; Srinisavan et al., 2012). The present 30% proportion of unsuccessful paediatric punctures complies well with the range from 7% to 69% reported in the literature (Auerbach et al., 2018; Baxter et al., 2006; Glatstein et al., 2011; Kessler et al., 2013; Neal et al., 2017 Nigrovic et al., 2007).

The target tissue of the bioimpedance needle, CSF, is derived from blood plasma and comprises mostly water (99%) with small electrolyte and glucose contents as well as subtle protein content. While different diseases and physiological conditions may modulate the chemical composition of CSF to some extent (Bonadio et al., 2014, Majumdar et al., 2013; Schiffer et al., 1999; Spector et al., 2015), the electrical conductivity of CSF is quite constant (about 1·8 S/m) showing small variation (standard deviation 1·2%) and being independent of age (Baumann et al., 1997). Further, the conductivity of CSF is twice that of human blood (Geddes & Baker, 1966), which makes CSF an ideal target for tissue classification with bioimpedance.

High spatial sensitivity and real‐time measurement provided by the bioimpedance needle system permit virtually an immediate detection of CSF when the tip of advancing needle reaches the subarachnoid space. Presumably, this functionality provides a means to prevent advancing the needle through subarachnoid space to its ventral wall and causing dural trauma and blood leakage into the spinal canal. The risk of dural trauma is known to increase with the number of attempts (Glatstein et al., 2011; Shah et al., 2003). On the other hand, given the high spatial sensitivity, it is possible that only the very tip of the bevelled needle is in contact with CSF at the time of the alarm, and when the stylet is removed for checking CSF flow, the needle tip may move backward while the dural strands block the lumen and the initial CSF contact disappears. This is a plausible explanation for the few false detections recorded in the CRFs, when the system gave an alarm, but there was no CSF flow after the stylet removal. This is an inherent pitfall for a spatially highly sensitive bioimpedance needle, but it may be avoided by carefully keeping the needle tip steady when the stylet is removed for checking CSF flow. Also, before removing the stylet, the needle may be rotated by 90° and slightly advanced as considered appropriate by the physician. Whether these actions would reduce false CSF detections remains to be assessed in further studies.

In the present study, haematological data were analysed from the third vial of the CSF sample according to hospital procedures, and if an adequate number of vials was not available because of technical or other problem (e.g. excessively bloody samples), priority was given to diagnostic bacteriological analysis. Those CSF samples from which the erythrocyte count was not determined, representing 30% of the procedures, may have contributed to the false detection rate as well. This is because the bioimpedance needle system can give an alarm also when the needle tip is in contact with a sufficiently conductive mix of CSF and blood. The alarm goes off when the classification algorithm finds that the measured impedance values reside sufficiently long time within the frequency‐specific ranges of impedance representing CSF. The highest evaluated erythrocyte count in the present study was 300 000 cells/µl, which indicates that approximately 1/10^th^ of the sample volume was blood, while the rest was likely CSF. This level of blood contamination would translate to a proportionally higher impedance, which remains marginal in relation to the fact that CSF is electrically ~ 100% more conductive fluid than blood in vessels (Baumann et al., 1997; Geddes & Baker, 1966). Given this large difference in conductivity, the system can distinguish CSF from blood – even in the presence of substantial blood contamination of CSF. If the bioimpedance needle system classified bloody CSF at the needle tip as CSF, but the laboratory abandoned the CSF sample because of being considered excessively blood‐tinged, one may rhetorically ask whether the detection made by the system was false.

Blood contamination to some extent was evident in almost half of the present CSF samples analysed in the laboratory. The visual threshold for blood in CSF is as low as about 400 erythrocytes/µl (Shah et al., 2003), a fact that makes the subjective visual evaluation of sample quality difficult. In the present study, 12 out of 28 analysed CSF samples could be considered bloody by visual assessment, and 10 (83%) of these were observed among neonates. Be it also noted that bloody CSF‐like fluid classified as CSF were obtained in eight of nine unsuccessful punctures, but the haematological analysis was not performed in the laboratory.

The present performance and safety findings from neonates and infants together with earlier findings from adults are promising and speak for the clinical feasibility of the novel bioimpedance needle system (Halonen et al., 2017). Knowing accurately in real‐time when the needle tip reaches CSF in the subarachnoid space may facilitate CSF sampling and intrathecal drug administration in clinical settings. Should the CSF detection functionality fail due to any reason, the mechanical performance of the bioimpedance needle as a spinal needle is not compromised and the lumbar puncture can be done as usual.

## 5 CONCLUSION

The novel bioimpedance needle system was found clinically feasible for lumbar punctures of neonates and infants. In those procedures, where a CSF sample was obtained and erythrocyte count was determined, representing 70% of all procedures, the CSF detection accuracy was 82%, whereas the false detection rate was 11%. The clinical utility of this novel method as an objective means to know the needle tip location in terms of the presence of CSF appears promising. Further clinical studies, preferably in a randomized controlled setting, are needed to demonstrate the benefit of the bioimpedance needle system.

## CONFLICT OF INTEREST

HS, JK, SH and TE are employees of Injeq Oy and hold stock options for tiny amounts of Injeq shares. Also, HS and TE own tiny amounts of Injeq shares. Other authors declare no conflicts of interest concerning this study, authorship and/or publication of this article.
